# MiR-98 modulates macrophage polarization and suppresses the effects of tumor-associated macrophages on promoting invasion and epithelial–mesenchymal transition of hepatocellular carcinoma

**DOI:** 10.1186/s12935-018-0590-3

**Published:** 2018-07-06

**Authors:** Lei Li, Pengfei Sun, Chengsheng Zhang, Zongchao Li, Kai Cui, Wuyuan Zhou

**Affiliations:** 1 Department of Hepatobiliary Surgery, Shandong Cancer Hospital Affiliated to Shandong University, Shandong Academy of Medical Sciences, Jinan, 250117 Shandong China; 2 Department of Hepatobiliary and Pancreatic Surgery, Xuzhou Cancer Hospital, No. 131 Huancheng Road, Gulou District, Xuzhou, 221000 Jiangsu China

**Keywords:** miR-98, Macrophage polarization, Tumor-associated macrophages, Epithelial–mesenchymal transition, Hepatocellular carcinoma

## Abstract

**Background:**

Tumor-associated macrophages (TAMs) are generally recognized as a promoter of tumor progression. miR-98 has been shown to suppress the proliferation, migration, invasion and epithelial–mesenchymal transition (EMT) of hepatocellular carcinoma (HCC) cells. Here, we aim to investigate the role of miR-98-mediated macrophage polarization in HCC progression.

**Methods:**

Human blood monocytes were isolated from healthy male donors and incubated with culture medium collected from HepG2 cells for 7 days. The phenotype of the macrophages was detected. The protein expression was detected by Western blot. Levels of cytokines secreted in culture medium were measured using the specific enzyme-linked immunosorbent assay kits. To explore the role of miR-98 in HCC-conditioned TAMs, HCC cells HepG2 and SMMC7721 were cultured with conditioned medium from HCC-conditioned TAMs that had been transfected with miR-98 mimic/inhibitor. Cell proliferation, migration and invasion assays were performed.

**Results:**

HCC-conditioned TAMs possessed M2-like phenotype, including increased protein expression of CD163 and TNF-α^low^, IL-1β^low^, TGF-β^high^ and IL-10^high^ phenotype. HCC-conditioned TAMs also promoted proliferation, migration, invasion and EMT of HepG2 and SMMC7721 cells. Furthermore, miR-98 modulated macrophage polarization from M2 to M1 in HCC-conditioned TAMs, as evidenced by the alteration of M1- or M2-related cytokines. Moreover, miR-98 mimic significantly suppressed the HCC-conditioned TAMs-mediated promotion of cell migration, invasion and EMT in HepG2 and SMMC7721 cells compared with negative control, whereas miR-98 inhibitor exerted reversed effects.

**Conclusions:**

miR-98 may play a vital role in regulating macrophage polarization, thereby suppressing the TAMs-mediated promotion of invasion and EMT in HCC.

**Electronic supplementary material:**

The online version of this article (10.1186/s12935-018-0590-3) contains supplementary material, which is available to authorized users.

## Background

Hepatocellular carcinoma (HCC) is one of the leading causes of cancer-related death worldwide, with 1 million deaths a year [1, 2]. Therefore, understanding of the molecular mechanisms underlying HCC is important for the development of effective treatment strategies. The tumor microenvironment is composed of cancer tissue and the surrounding stromal cells, and provides opportunity for reciprocal interactions among inflammatory cells, cancer cells and microcapillary vessels [3]. Interestingly, HCC is one of inflammation-related cancers, as the chronic inflammatory state appears to be necessary for the initiation and development of liver cancer [4].

Macrophages, highly plastic cells, can be polarized to achieve a spectrum of functional phenotypes. In response to a variety of microenvironmental stimuli [5]. Pro-inflammatory M1 macrophages show tumoricidal activity and promote T helper (Th) 1 responses, whereas M2 macrophages display regulatory functions in tissue repair and remodeling, and promote Th2 immune responses [6]. In addition, M1 macrophages can enhance cell recruitment to the inflammatory focus by secreting tumor necrosis factor (TNF)-α, interleukin (IL)-1β, and nitric oxide. Furthermore, M2 macrophages enhance fungal phagocytosis and secrete pro-resolution substances including fibronectin, IL-10, transforming growth factor (TGF)-β, and metalloproteases [7]. Tumor-associated macrophages (TAMs) mostly show an M2-like phenotype [8]. TAMs are key orchestrators of the tumor microenvironment directly affecting neoplastic cell growth, neoangiogenesis, and extracellular matrix remodeling [9]. Indeed, increased infiltration of TAMs has been associated with poor prognosis and worse pathological characteristics in several cancers, including breast cancer, colon cancer, bladder cancer, prostate carcinoma, and also HCC [3, 10–12].

MicroRNAs (miRNAs) can regulate 30–90% of human genes and play important roles in cell growth, activation, apoptosis and differentiation [3]. miR-98, belongs to the let-7 family, acts as an oncogene or tumor suppressor in some human cancers [13, 14]. miR-98 has been shown to inhibit HCC proliferation via targeting enhancer of zeste homolog-2 (EZH2) and suppressing Wnt/β-catenin signaling pathway [15]. Recently, Lin-28B was shown to promote tumor formation and invasion in HCC through coordinated repression of the let-7/miR-98 family and induction of multiple oncogenic pathways [16]. Our previous study demonstrated that miR-98 played a suppressive role in the proliferation, migration, invasion and epithelial–mesenchymal transition (EMT) of HCC cells via targeting Sal-like protein 4 [17]. Recent studies have shown that let-7b, another member of the let-7 family, was up-regulated in prostatic TAMs and modulated macrophage polarization; and the decreased expression of let-7b inhibited the pro-angiogenic effect of TAMs and their capacity to enhance prostate carcinoma cell motility [3]. Therefore, we hypothesized that miR-98, another member of the let-7 family, may also modulate macrophage polarization and affect the effects of TAMs on the invasion and EMT of HCC. This study investigated the effects of miR-98 in regulating macrophage polarization and explored the role of miR-98-mediated macrophage polarization in HCC progression.

## Methods

### Isolation and culture of human peripheral blood macrophages

Blood monocytes were isolated from healthy donor buffy coats. Peripheral blood mononuclear cells (PBMCs) were isolated using a Ficoll (Solarbio Life Sciences, Beijing, China) density gradient. Monocytes were purified with anti-CD14 paramagnetic beads (Miltenyi Biotec, Auburn, CA), according to the manufacturer’s instruction. Non-adherent cells were removed, and the purity of monocytes (> 95%) was determined by flow cytometric analysis. CD14^+^monocytes (5 × 10^5^cells/ml) were cultured with RPMI 1640 (Sigma Co., St. Louis, Mo., USA), supplemented with 10% fetal bovine serum (FBS; Sigma Co., St. Louis, Mo., USA) and 50 ng/ml macrophage colony-stimulating factor (M-CSF, Sigma Co., St. Louis, Mo., USA) for 7 days. Half of culture medium was changed every 3 days, unless otherwise indicated. To obtain M0 cells, CD14^+^monocytes were treated with serum-free medium for 48 h. To polarize M1 macrophages, cells were stimulated overnight with 100 ng/ml lipopolysaccharides (LPS, Sigma Co., St. Louis, Mo., USA) and 100 ng/ml IFN-γ (Sigma Co., St. Louis, Mo., USA). To polarize M2 macrophages, cells were stimulated overnight with 20 ng/ml IL-4 (Sigma Co., St. Louis, Mo., USA). TAMs of HCC were obtained by culturing monocytes isolated from PBMCs for 7 days in RPMI 1640 containing 10% FBS with 50% of conditioned medium from HepG2 cells. Conditioned medium was obtained from untreated HepG2 cells. M0, M1, M2 and TAMs cells were incubated for 48 h in serum-starved condition, and culture medium was harvested and clarified by centrifugation and used freshly. Written informed consent was obtained from all donors. The study was approved by the Ethics Committee of Shandong Cancer Hospital Affiliated to Shandong University.

### Quantification of cytokine levels

Levels of cytokines including TNF-α, IL-1β, TGF-β and IL-10 secreted in culture medium were measured using the specific enzyme-linked immunosorbent assay (ELISA) system kits (Abcam, Chicago, IL USA), according to the manufacturer’s instructions.

### Cell culture

Human HCC cell lines HepG2 and SMMC7721 were purchased from the Chinese Academy of Sciences (Shanghai, China). Cells were cultured in Dulbecco’s modified Eagle’s medium (DMEM, Life Technologies), supplemented with 10% FBS, 100 IU/ml penicillin and 100 IU/ml streptomycin. Cells were cultured at 37 °C in a humidified atmosphere with 5% CO_2_. Cells were then incubated with culture medium of M0, M1, M2, TAMs cells or 1640 medium as control for 48 h. The culture medium of cells was harvested and clarified by centrifugation and used freshly.

### MTT assay

The 3-(4, 5-dimethylthiazal-2-yl)-2, 5-diphenyl-tetrazolium bromide (MTT) assay was used to examine cell proliferation as described in our previous study [17]. Briefly, cells in each group were plated at a density of 1 × 10^4^ cells per well in 96-well plates. Cells were then incubated with MTT at a final concentration of 0.5 mg/ml for 4 h at 37 °C. After the removal of the medium, 150 mM DMSO solutions were added to dissolve the formazan crystals. The absorbance was measured at 570 nm using a Bio-Tek™ ELX-800™ Absorbance Microplate reader (Bio-Tek Instruments Inc., USA).

### Cell migration assay

Wound healing assay was performed to evaluate the cell migratory capacity of HCC cells as described in our previous study [17]. In brief, cells were cultured to reach 70–80% confluence. Wounds of approximately 1 mm width were created with a plastic scriber, and cells were washed and incubated in a serum-free medium. At 24 h after wounding, cells were incubated in a medium supplemented with 10% FBS. After 48 h of culture, cells were fixed and observed under a microscope (original magnification, 100×; Olympus, Tokyo, Japan).

### Cell invasion assay

Cell invasion assay was performed using Transwell chambers (BD Biosciences, Bedford, Massachusetts, USA) as described in our previous study [17]. Cells were pre-coated with Matrigel. Cell suspension containing 5 × 10^5^ cells/ml was prepared in serum-free media, and 300 μl cell suspension was added into the upper chamber. Then, 500 μl DMEM with 10% FBS was added into the lower chamber. Cells were incubated for 24 h. Then, we used a cotton-tipped swab to carefully wipe out the cells that did not invade through the pores. The filters were fixed in 90% alcohol and stained by crystal violet, and observed under an inverted microscope (original magnification, 100×; Olympus, Tokyo, Japan).

### miR mimic and inhibitor

The miR-98 mimic, mimic negative control (NC), miR-98 inhibitor and inhibitor NC were purchased from Amspring (Changsha, China), and were transfected into M0, M1, M2 and TAMs cells by Lipefectamin-2000.

### Western blot

Western blot was performed as described in our previous study [17]. Cells were lysed with ice-cold lysis buffer (50 mM Tris–HCl, pH6.8, 100 mM 2-ME, 2% w/v SDS, 10% glycerol). After centrifugation at 20,000×*g* for 10 min at 4 °C, proteins in the supernatants were quantified and separated with 10% SDS-PAGE. Then, proteins were transferred onto a polyvinylidene difluoride (PVDF) membrane (Amersham Bioscience, Buckinghamshire, USA), which was then incubated with PBS containing 5% milk overnight at 4 °C. The PVDF membrane was incubated with rabbit anti-human primary antibodies: CD163 (1:1000, ab17051), E-cadherin (1:1000, ab15148), N-cadherin (1:1000, ab18203), Fibronectin (1:1000, ab2413), vimentin (1:1000, ab16700) and GAPDH (1:10,000, ab181602) (all from Abcam, Cambridge, MA, USA) at room temperature for 3 h, respectively, and then incubated with mouse anti-rabbit secondary antibody (1:10,000, ab99702, Abcam) at room temperature for 1 h. Super Signal West Pico Chemiluminescent Substrate Kit (Pierce, Rockford, IL, USA) was then used to detect signals, according to the manufacture’s instruction. The relative protein expression was analyzed by Image-Pro plus software 6.0, represented as the density ratio versus GAPDH.

### RNA extraction and real-time reverse transcription PCR

Total RNA was extracted using Invitrogen Trizol Reagent (Life Technologies Corporation). For miRNA quantification, 100 ng total RNA was reverse transcribed directly using stem-loop primers. Quantitative real-time PCR was performed using the SYBR Green PCR Master Mix (Tokara, Kyoto, Japan) in a final volume of 20 μl on Bio-RAD CFX96 TM Real-Time System (Bio-Rad Laboratories, Inc., Hercules, CA, USA). The expression of miRNA was normalized to U6. Data are presented as relative quantification based on the calculation of 2^−ΔΔCt^.

### Statistical analysis

SPSS16.0 software was used for statistical analysis. All data were presented as mean ± standard deviation (SD) of three independent experiments. The error bars in each figure represent SD of three independent experiments. One-way analysis of variance (ANOVA) was used for comparison. P < 0.05 was considered to indicate a statistically significant difference.

## Results

### Characterization of HCC-conditioned TAMs

**Fig. 1:**
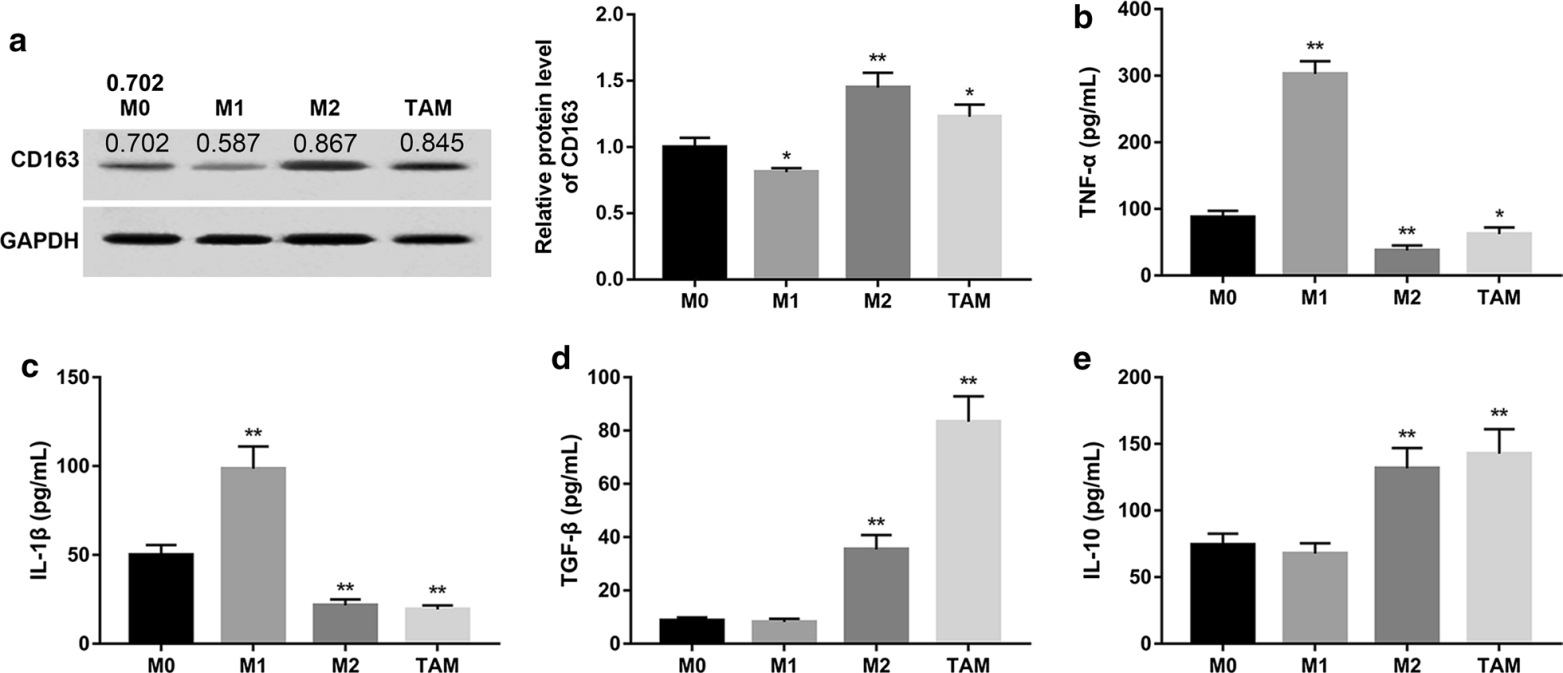
Characterization of HCC-conditioned TAMs. Human monocytes were isolated from normal donor buffy coat using anti-CD14 magnetic beads. Monocytes were cultured in the presence of M-CSF for 7 days. TAMs, M1 and M2 macrophages were differentiated as described in “Methods”. **a** The protein expression of CD163 in each group was examined by Western blot. Densitometric quantification was shown. GAPDH served as the loading control. Levels of cytokines including TNF-α (**b**), IL-1β (**c**), TGF-β (**d**) and IL-10 (**e**) secreted in culture medium were measured using the specific ELISA system kits. *P < 0.05 and **P < 0.01 vs. the M0 group

Circulating monocytes can pass through the vascular endothelium to mature into macrophages in the peripheral tissues and are activated in various ways through endogenous and exogenous factors. To investigate whether exposure to HCC tumor microenvironment affected monocyte differentiation, human blood monocytes were incubated with culture medium collected from HepG2 cells for 7 days, and the phenotype of the macrophages were detected (Additional file 1: Figure S1). Data revealed that the protein expression of CD163, a marker for M2, was significantly decreased in M1 group compared with the M0 group (Fig. 1a). On the contrary, the protein expression of CD163 was significantly increased in M2 and TAM group compared with the M0 group. Next, we measured the expression of TNF-α, IL-1β, TGF-β and IL-10, which have been used for phenotyping macrophages. Our results showed that both M2 macrophages and TAMs displayed TNF-α^low^, IL-1β^low^, TGF-β^high^ and IL-10^high^ phenotype (Fig. 1b–e). These data indicated that HCC-conditioned TAMs possessed M2-like phenotype.

### HCC-conditioned TAMs promote proliferation, migration, invasion and EMT of HepG2 and SMMC7721 cells in vitro

**Fig. 2:**
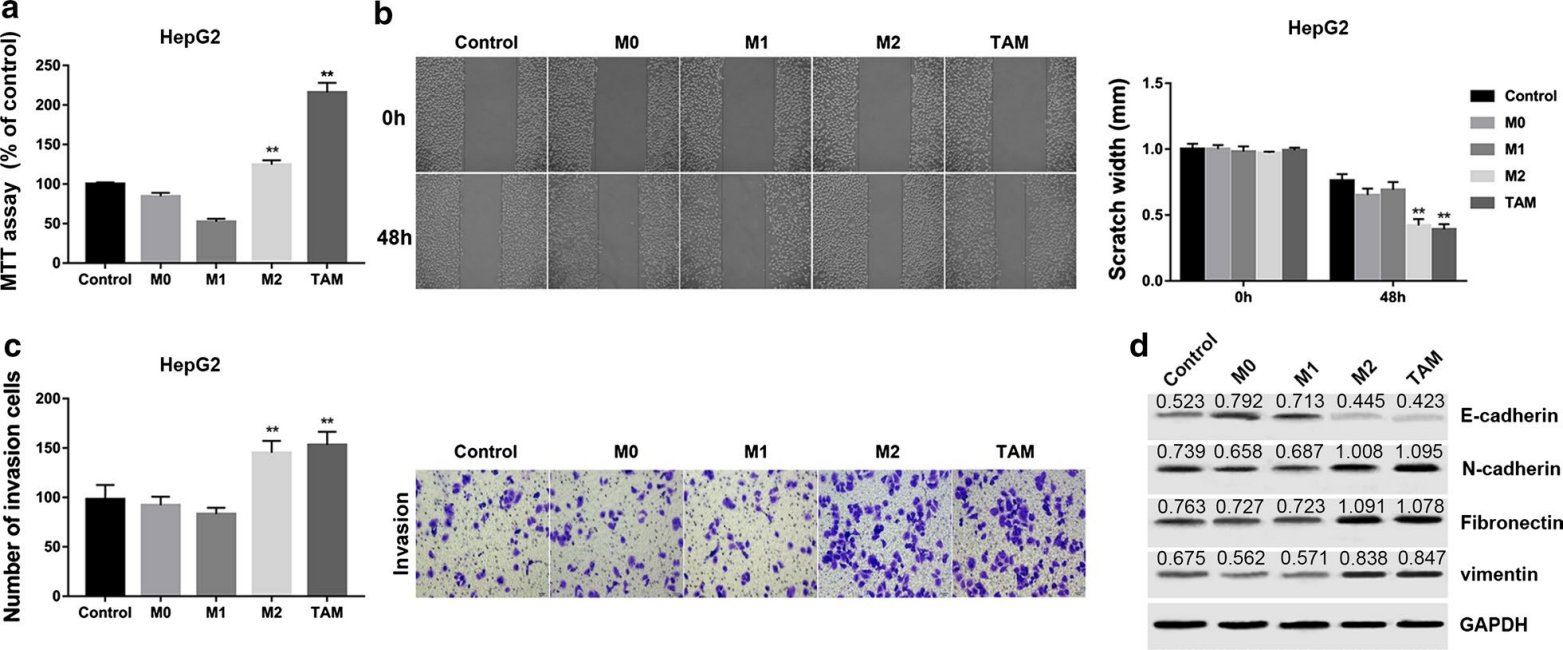
HCC-conditioned TAMs promote proliferation, migration, invasion and EMT of HepG2 cells in vitro. HepG2 cells were incubated with culture medium of M0, M1, M2 and TAMs cells for 48 h. **a** MTT assay showed that TAMs promoted proliferation of HepG2 cells in vitro. **b** Wound healing assay demonstrated that TAMs promoted migration of HepG2 cells in vitro. **c** Transwell invasion assay showed that TAMs promoted invasion of HepG2 cells in vitro. Data are presented as the mean number of the migration and invasion cells per filed counted from five randomly selected fields under a microscope (×100 magnification). **d** Western blot analysis of EMT-related proteins showed that TAMs decreased the expression of E-cadherin but increased the protein levels of N-cadherin, fibronectin and vimentin, indicating that TAMs promoted EMT of HepG2 cells in vitro. Densitometric quantification was shown. **P < 0.01 vs. the control group

**Fig. 3:**
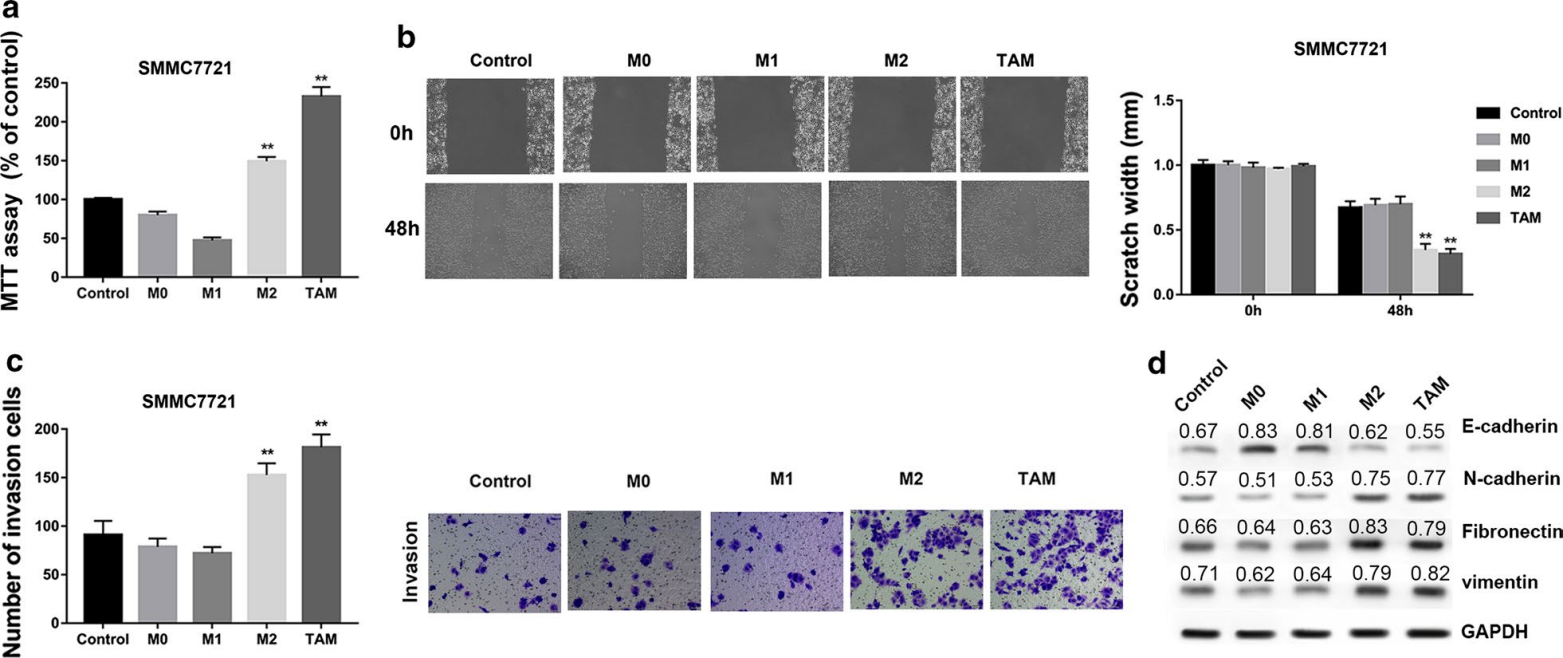
HCC-conditioned TAMs promote proliferation, migration, invasion and EMT of SMMC7721 cells in vitro. SMMC7721 cells were incubated with culture medium of M0, M1, M2 and TAMs cells for 48 h. **a** MTT assay showed that TAMs promoted proliferation of SMMC7721 cells in vitro. **b** Wound healing assay demonstrated that TAMs promoted migration of SMMC7721 cells in vitro. **c** Transwell invasion assay showed that TAMs promoted invasion of SMMC7721 cells in vitro. Data are presented as the mean number of the migration and invasion cells per filed counted from five randomly selected fields under a microscope (×100 magnification). **d** Western blot analysis of EMT-related proteins showed that TAMs decreased the expression of E-cadherin but increased the protein levels of N-cadherin, fibronectin and vimentin, indicating that TAMs promoted EMT of SMMC7721 cells. Densitometric quantification was shown. **P < 0.01 vs. the control group

Given that HCC-conditioned TAMs possessed M2-like phenotype, we next analyzed whether these TAMs possessed the M2-like effects on HepG2 cells in vitro. HepG2 cells were then incubated with culture medium of M0, M1, M2 and TAMs cells or 1640 medium as control for 48 h. Subsequently, we evaluated the proliferative, migratory and invasive variation of HepG2 cells. We found that HepG2 cells incubated with TAMs showed a remarkable promotion in cell proliferation (Fig. 2a), invasion (Fig. 2b), and migration (Fig. 2c) compared with the control HepG2 cell group. Furthermore, the TAMs group showed decreased protein levels of E-cadherin but increased levels of N-cadherin, fibronectin and vimentin, indicating that EMT was induced by TAMs (Fig. 2d). These effects were comparable to the results observed in HepG2 cells that incubated with culture medium of M2 macrophages. Also, similar results were observed in SMMC7721 cells (Fig. 3).

### miR-98 regulates macrophage polarization in HCC-conditioned TAMs

**Fig. 4:**
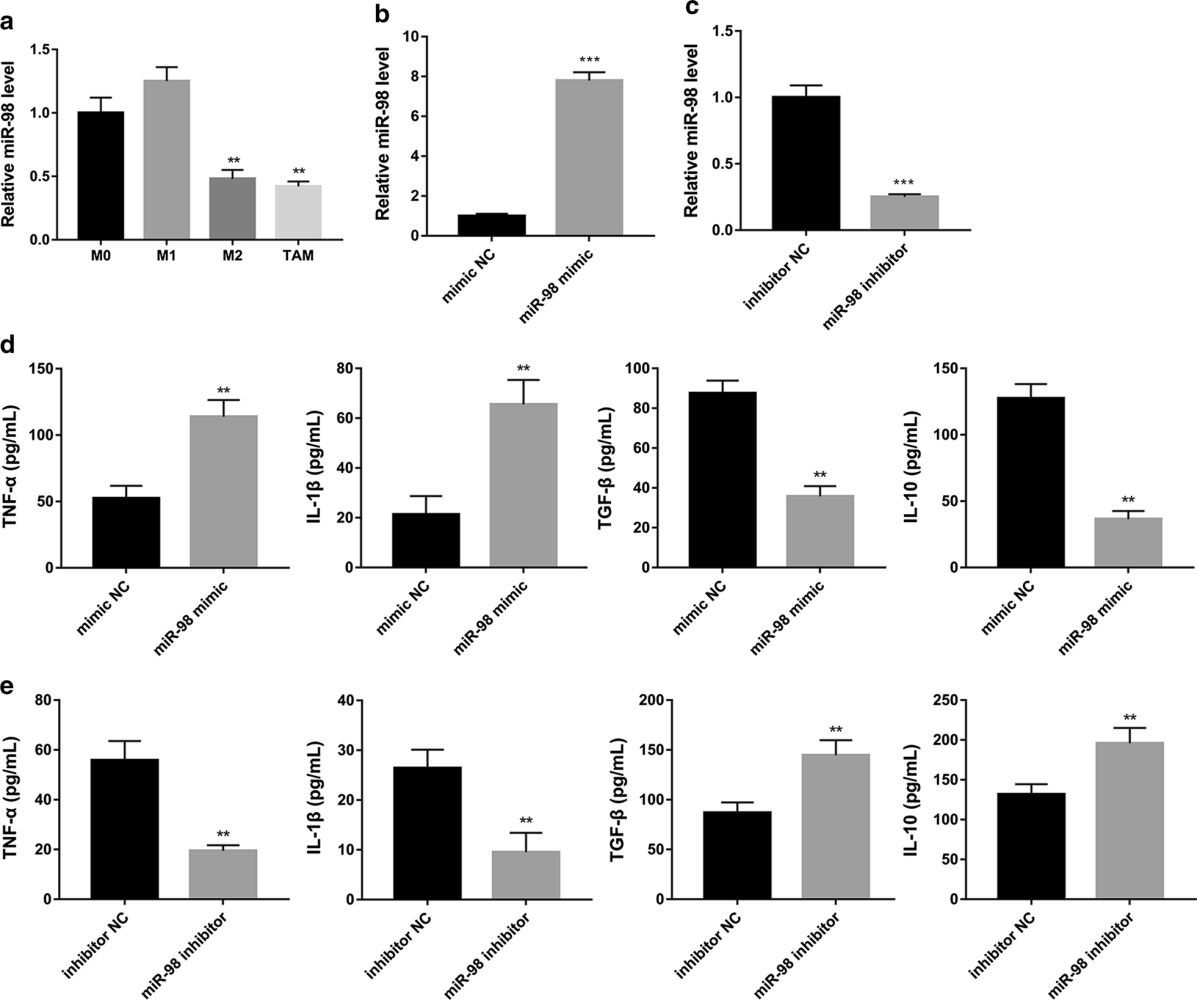
MiR-98 regulates expression of inflammatory cytokines in HCC-conditioned TAMs. **a** The expression levels of miR-98 in M2 and TAMs were significantly decreased compared with the M0 group. Next, TAMs were transfected with miR-98 mimic, mimic NC, miR-98 inhibitor or inhibitor NC. Quantitative real-time PCR showed that miR-98 level **b** in TAMs was significantly up-regulated by miR-98 mimic, and **c** was significantly down-regulated by miR-98 inhibitor. **d** ELISA assay showed that the levels of TNF-α and IL-1β were significantly increased whereas the levels of TGF-β and IL-10 were significantly decreased in HCC-conditioned TAMs that had been transfected with miR-98 mimic compared with the control group. **e** ELISA assay showed that the levels of TNF-α and IL-1β were significantly decreased whereas the levels of TGF-β and IL-10 were significantly increased in HCC-conditioned TAMs that had been transfected with miR-98 inhibitor compared with the control. **P < 0.01 and ***P < 0.001 vs. the corresponding control group

Next, we detected the expression of miR-98 in M0, M1, M2, and TAMs. We found that the expression levels of miR-98 in M2 and TAMs were significantly decreased compared with the M0 group (Fig. 4a). After that, we transfected TAMs with miR-98 mimic, mimic NC, miR-98 inhibitor and inhibitor NC to investigate whether miR-98 regulated macrophage polarization in HCC-conditioned TAMs. We found that miR-98 level was significantly up-regulated by miR-98 mimic (Fig. 4b) and significantly down-regulated by miR-98 inhibitor (Fig. 4c), compared with the corresponding controls. It has been reported that TAMs exhibited two different patterns of phenotype, M1 or M2, depending on their activation pathway [18]. M1 macrophages secrete TNF-α and IL-1β, and M2 macrophages secrete TGF-β and IL-10 [7]. Our results showed that, in the presence of miR-98 mimic, the levels of TNF-α and IL-1β were significantly increased whereas the levels of TGF-β and IL-10 were significantly decreased in HCC-conditioned TAMs (Fig. 4d). However, the levels of TNF-α and IL-1β were significantly decreased whereas the levels of TGF-β and IL-10 were significantly increased in HCC-conditioned TAMs that transfected with miR-98 inhibitor compared with the control (Fig. 4e). These results indicated that miR-98 may modulate macrophage polarization from M2 to M1 in HCC-conditioned TAMs.

### HCC-conditioned TAMs treated with miR-98 regulate migration, invasion and EMT of HepG2 and SMMC7721 cells

**Fig. 5:**
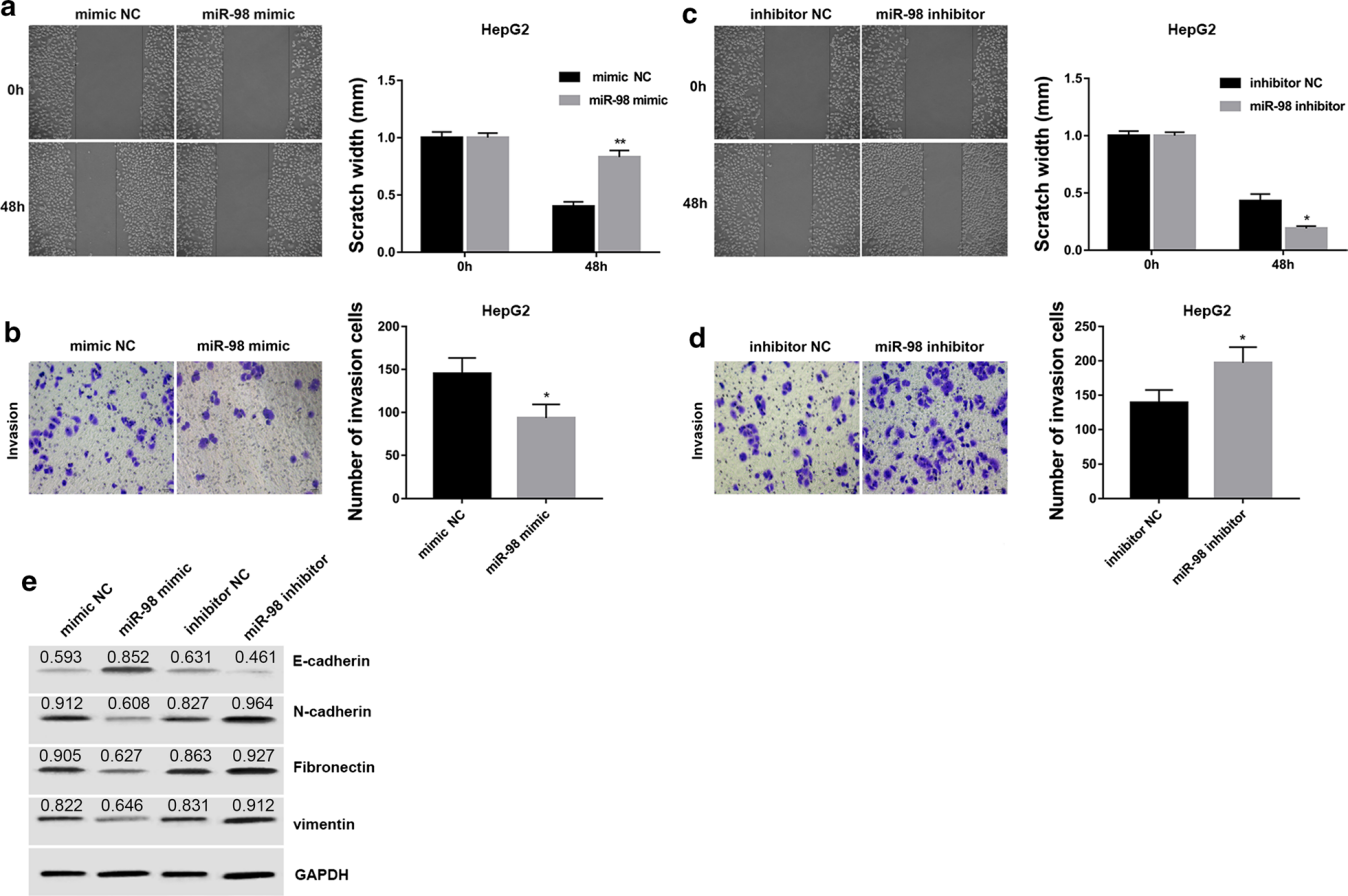
HCC-conditioned TAMs treated with miR-98 regulate migration, invasion and EMT of HepG2 cells. HepG2 cells were cultured and scraped away using a pipette tip. Subsequently, cells were cultured with conditioned medium from TAMs treated with miR-98 mimic, mimic NC, miR-98 inhibitor and inhibitor NC for 48 h. miR-98 mimic significantly suppressed the capacity of HCC-conditioned TAMs to promote HepG2 cell migration (**a**) and invasion (**b**) compared with negative control. miR-98 inhibitor significantly enhanced the capacity of HCC-conditioned TAMs to promote HepG2 cell migration (**c**) and invasion (**d**) compared with negative control. Wound healing assay was performed to evaluate the migratory capacity of HCC cells. Cell invasion assay was performed using Transwell chambers. Data are presented as the mean number of the migration and invasion cells per filed counted from five randomly selected fields under a microscope (×100 magnification). **e** miR-98 mimic suppressed the capacity of HCC-conditioned TAMs to promote HepG2 cell EMT while miR-98 inhibitor enhanced the capacity of HCC-conditioned TAMs to promote HepG2 cell EMT compared with negative control. Densitometric quantification was shown. *P < 0.05, **P < 0.01 vs. the corresponding control group

**Fig. 6:**
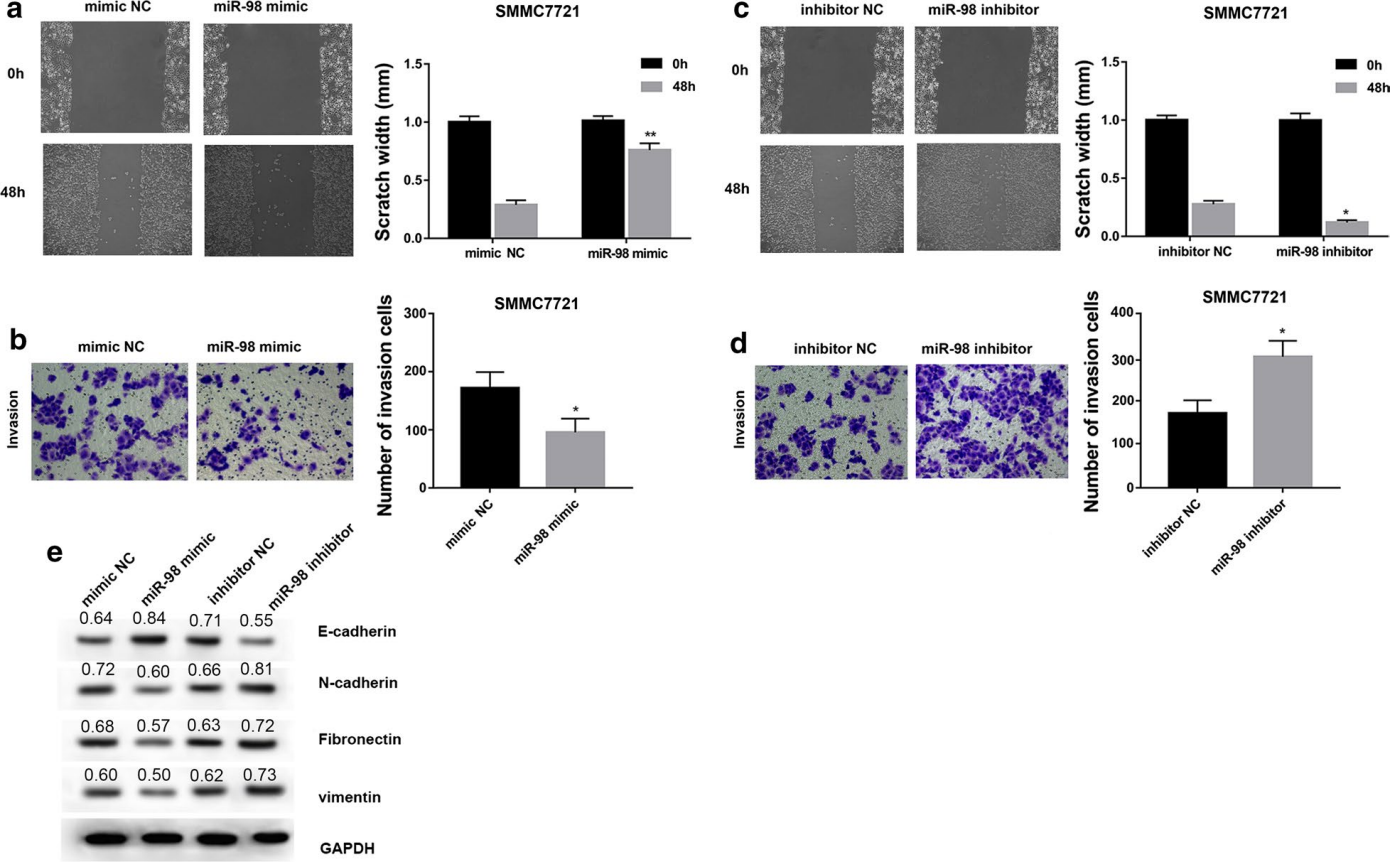
HCC-conditioned TAMs treated with miR-98 regulate migration, invasion and EMT of SMMC7721 cells. SMMC7721 cells were cultured and scraped away using a pipette tip. Subsequently, cells were cultured with conditioned medium from TAMs treated with miR-98 mimic, mimic NC, miR-98 inhibitor and inhibitor NC for 48 h. miR-98 mimic significantly suppressed the capacity of HCC-conditioned TAMs to promote SMMC7721 cell migration (**a**) and invasion (**b**) compared with negative control. miR-98 inhibitor significantly enhanced the capacity of HCC-conditioned TAMs to promote SMMC7721 cell migration (**c**) and invasion (**d**) compared with negative control. Wound healing assay was performed to evaluate the migratory capacity of HCC cells. Cell invasion assay was performed using Transwell chambers. Data are presented as the mean number of the migration and invasion cells per filed counted from five randomly selected fields under a microscope (×100 magnification). **e** miR-98 mimic suppressed the capacity of HCC-conditioned TAMs to promote SMMC7721 cell EMT while miR-98 inhibitor enhanced the capacity of HCC-conditioned TAMs to promote SMMC7721 cell EMT compared with negative control. Densitometric quantification was shown. *P < 0.05, **P < 0.01 vs. the corresponding control group

To clarify whether introduction of miR-98 mimic/inhibitor into TAMs could impair HepG2 and SMMC7721 cell growth, we incubated human HepG2 and SMMC7721 cells with culture medium from TAMs that had been transfected with miR-98 mimic/inhibitor or the corresponding controls. Data revealed that the capacity of HCC-conditioned TAMs to promote cell migration and invasion of HepG2 and SMMC7721 cells was significantly suppressed by miR-98 mimic but enhanced by miR-98 inhibitor compared with their corresponding control (Figs. 5a–d, and 6a–d). Furthermore, the capacity of HCC-conditioned TAMs to promote EMT of HepG2 and SMMC7721 cells was suppressed by miR-98 mimic but enhanced by miR-98 inhibitor compared with their corresponding control (Figs. 5e and 6e). These results suggested that miR-98 expression in HCC-conditioned TAMs played a critical role in regulating migration, invasion and EMT of HepG2 and SMMC7721 cells.

## Discussion

TAMs have been associated with enhanced tumor progression, including cancer cell growth and immune suppression. In this study, human monocytes became HCC-conditioned TAMs after incubation with conditioned medium collected from HepG2 cell culture. The resultant TAMs displayed characteristics of M2-like macrophages, including increased protein expression of CD163, TNF-α^low^, IL-1β^low^, TGF-β^high^ and IL-10^high^ phenotype. The up-regulation of M2-associated CD163 in HCC-infiltrating macrophages has been recently demonstrated [19]. Moreover, an established M2 macrophage population has been associated with poor prognosis in HCC [20]. Here, we found that TAMs significantly enhanced cell migration, invasion, and EMT in HepG2 and SMMC7721 cells in vitro compared with the control macrophages. Our results indicate the important role of HCC-conditioned TAMs in HepG2 and SMMC7721 cells. However, inconsistent with the pro-tumor effects of TAMs in our study, other studies suggested that the infiltration of TAMs could protect HCC patients from recurrence and metastasis, and that high levels of pro-inflammatory molecules derived from tumor-infiltrating cells were associated with a better survival in HCC patients [21, 22]. The contradictory results may be attributed to several factors such as the difference between in vivo and in vitro environment, number of samples, and different experimental procedures.

miRNAs are universal regulators of differentiation, activation and polarization of macrophages. For example, Chaudhuri et al. [23] suggested that miR-125b is responsible for generating the activated nature of macrophages and potentiating the functional role of macrophages in inducing immune responses. Androulidaki et al. [24] indicated that the kinase Akt1 controls macrophage response to lipopolysaccharide by regulating microRNAs, such as let-7e, miR-181c, miR-155 and miR-125b. Kumar et al. [25] showed that let-7f, another member of the let-7 family, was over-expressed in tuberculosis-infected macrophages that induced TNF and IL-1β secretion. A recent study demonstrated that miRNA let-7b modulates macrophage polarization and the decreased expression of let-7b inhibits the pro-angiogenic effect of TAMs and their capacity to enhance prostate carcinoma cell motility [3]. However, the role of miR-98, another member of the let-7 family, in modulating macrophage polarization, has not yet been defined. Our results showed that miR-98 is expressed in HCC-conditioned TAMs at the lower level compared with M0 and M1 macrophages. Furthermore, miR-98 mimic significantly increased levels of TNF-α and IL-1β (secreted by M1 macrophages) but decreased levels of TGF-β and IL-10 (secreted by M2 macrophages) in HCC-conditioned TAMs. In contrast, miR-98 inhibitor exerted the opposite effects on these cytokines. These results indicate that miR-98 regulates macrophage polarization from M2 to M1 in HCC-conditioned TAMs.

Some studies have demonstrated that miR-98 has suppressive effects on several cancers, such as oral squamous cell carcinoma, non-small-cell lung cancer, glioma, melanoma and HCC [17, 26–29]. A recent study demonstrated that the CCL18-mediated down-regulation of miR-98 enhanced the EMT of breast cancer cells, and thus promoted breast cancer metastasis [30]. In this study, to explore the role of miR-98 in HCC-conditioned TAMs, HepG2 and SMMC7721 cells were cultured with conditioned medium from TAMs that were treated with miR-98 mimic or miR-98 inhibitor for 48 h. We found that miR-98 not only regulated expression of inflammatory cytokines in HCC-conditioned TAMs, but also suppressed the capacity of HCC-conditioned TAMs to promote HepG2 and SMMC7721 cell migration, invasion, and EMT. One plausible explanation might be that miR-98 regulates macrophage polarization from M2 to M1 in HCC-conditioned TAMs. Furthermore, a previous study demonstrated that the TAM-released cytokines and chemokines play a vital role in the initiation and progression of liver cancer and regulation of tumor growth, invasion, and metastasis [4]. Collectively, the miR-98-mediated macrophage polarization from M2 to M1 and regulation of cytokines may lead to the change in biological properties of TAMs.

It is well-established that miRNAs inhibit protein expression by binding to the 3’-untranslated region of target mRNAs, leading to transcriptional repression or degradation of the mRNA. By using TargetScan and PicTar, several HCC-related genes (such as IL-10, LIN28B, HMGA2) [16, 31] were identified as the potential targets of miR-98. Therefore, we speculate that these potential targets may involve in the miR-98-mediated regulation in macrophage polarization in HCC-conditioned TAMs and in the capacity of HCC-conditioned TAMs to regulate HCC cell migration and invasion, which needs our further investigation.

## Conclusions

In conclusion, our findings showed that miR-98 may play a vital role in regulating macrophage polarization, thus suppressing the capacity of TAMs to promote invasion and EMT of HCC. Our study validated the vital role of miR-98-mediated macrophage polarization in HCC progression. It is our expectation that additional target genes of miR-98, such as IL-10, LIN28B, HMGA2, will be identified in the future. Our results suggest that miR-98 is a promising modulator for macrophage polarization and may become a promising therapeutic target for HCC treatment.

## Additional file

**Additional file 1: Figure S1.** The differentiation proportion of different types of macrophages. (A) Human monocytes were isolated from PBMCs by sorting with anti-CD14 magnetic beads. Macrophages were prepared from these monocytes by culture for 7 days in RPMI 1640 medium containing 10% FBS in the presence of 50 ng/ml M-CSF. Flow cytometry revealed that the purified cells were >95% CD14^+^ cells. (B) To obtain M0 cells, CD14^+^monocytes were treated with serum-free medium for 48 h. To polarize M1 macrophages, macrophages were stimulated overnight with 100 ng/ml LPS, and 100 ng/ml IFN-γ. To polarize M2 macrophages, macrophages were stimulated with overnight with 20 ng/ml IL-4. The differentiation proportion of M0 (CD16/23^-^CD206^-^), M1 (CD16/23^+^) and M2 (CD206^+^) macrophages detected by flow cytometry was 87%, 89% and 96%, respectively.

## Abbreviations

ELISA: enzyme-linked immunosorbent assay
EMT: epithelial–mesenchymal transition
HCC: hepatocellular carcinoma
IL-1β: interleukin-1β
miRNAs: microRNAs
PBMCs: peripheral blood mononuclear cells
TAMs: tumor-associated macrophages
TGF-β: transforming growth factor-β
Th: T helper
TNF-α: tumor necrosis factor-α
