# Supplementary figures and images for: MiR-98 modulates macrophage polarization and suppresses the effects of tumor-associated macrophages on promoting invasion and epithelial–mesenchymal transition of hepatocellular carcinoma
Source: Cancer Cell Int. 2018 Jul 6;18:95. doi: 10.1186/s12935-018-0590-3 (PMC6035433; doi:10.1186/s12935-018-0590-3)

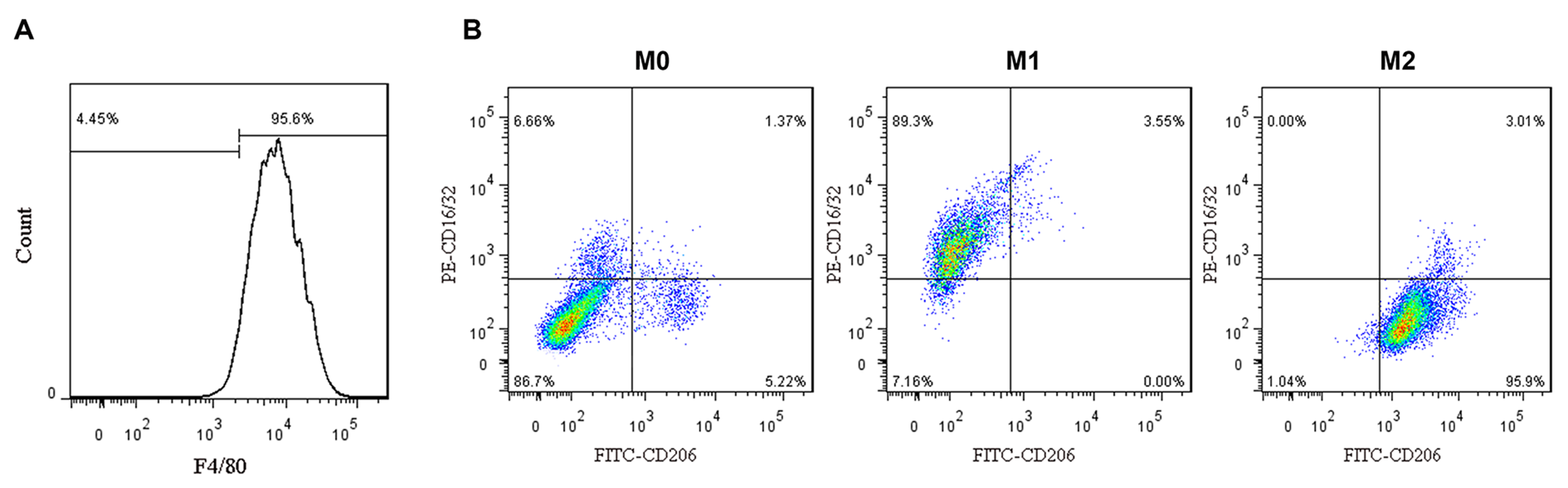

Supplement: Supplementary file 1 — Additional file 1: Figure S1. The differentiation proportion of different types of macrophages. (A) Human monocytes were isolated from PBMCs by sorting with anti-CD14 magnetic beads. Macrophages were prepared from these monocytes by culture for 7 days in RPMI 1640 medium containing 10% FBS in the presence of 50 ng/ml M-CSF. Flow cytometry revealed that the purified cells were >95% CD14+ cells. (B) To obtain M0 cells, CD14+monocytes were treated with serum-free medium for 48 h. To polarize M1 macrophages, macrophages were stimulated overnight with 100 ng/ml LPS, and 100 ng/ml IFN-γ. To polarize M2 macrophages, macrophages were stimulated with overnight with 20 ng/ml IL-4. The differentiation proportion of M0 (CD16/23-CD206-), M1 (CD16/23+) and M2 (CD206+) macrophages detected by flow cytometry was 87%, 89% and 96%, respectively. [file 12935_2018_590_MOESM1_ESM.tif]
